# Nonalcoholic Fatty Liver Disease Is Associated with *Helicobacter pylori* Infection in North Urban Chinese: A Retrospective Study

**DOI:** 10.1155/2020/9797841

**Published:** 2020-01-25

**Authors:** Mei-Yan Xu, Jia-Hui Ma, Juan Du, Jian Yin, Lan Liu, Fuqiang Cui, Qing-Bin Lu

**Affiliations:** ^1^Department of Nutrition, Aerospace Center Hospital, Beijing 100049, China; ^2^Department of Laboratorial Science and Technology, School of Public Health, Peking University, Beijing 100191, China; ^3^Department of Health Management, Aerospace Center Hospital, Beijing 100049, China; ^4^Beijing Key Laboratory of Toxicological Research and Risk Assessment for Food Safety, Beijing 100191, China

## Abstract

**Background:**

The association between nonalcoholic fatty liver disease (NAFLD) and *Helicobacter pylori* (*H. pylori*) is controversial. We conducted a retrospective study to clarify the seroprevalence of *H. pylori* infection and the relationship between NAFLD and *H. pylori* infection in north urban Chinese.

**Methods:**

The retrospective study was performed at Aerospace Center Hospital in Beijing. All subjects in this study were a healthy population who underwent health examinations at the hospital between 2012 and 2015. A logistic regression model was used to calculate the association between NAFLD and *H. pylori* infection. Age, gender, underlying diseases, and metabolic syndrome (MS) were adjusted. Effects of NAFLD on *H. pylori* infection in a different age, gender, and number of MS characteristic subgroups were analyzed.

**Results:**

There were 7803 (43.4%) subjects with *H. pylori* infection, 3726 (20.7%) with mild NAFLD, 730 (4.1%) with moderate NAFLD, and 369 (2.1%) with severe NAFLD among 17971 subjects. *H. pylori* infection was related to the seroprevalence of any level of NAFLD, including mild, moderate, and severe NAFLD (OR = 1.607, 95% CI: 1.487-1.736; OR = 1.770, 95% CI: 1.519-2.063; and OR = 2.120, 95% CI: 1.714-2.526, respectively). The results of subgroup analysis showed that the risk of incident NAFLD from *H. pylori* infection had significant interactions by subjects with or without MS characteristics. Moreover, as the number of MS characteristics in patients with a fatty liver increased, the risk of *H. pylori* infection also increased.

**Conclusions:**

NAFLD may be associated with *H. pylori* infection in a Chinese population. Younger, male NAFLD patients and those meeting more characteristics of MS were more likely to have *H. pylori* infection.

## 1. Introduction

Nonalcoholic fatty liver disease (NAFLD) is one of the most prevalent chronic liver diseases in the world. It is believed to be the manifestation of metabolic syndrome (MS) in the liver and is caused by a variety of factors such as nutritional disorders, dyslipidemia, insulin resistance, and genetic factors [1]. According to reports, NAFLD affects 20%-45% of the general population and 60%-75% of obese people [2, 3], and the incidence of NAFLD is rapidly increasing, causing a great clinical and economic burden [3]. Some reports mentioned the relationship between NAFLD and intestinal microbes [4]. Since the “gut-liver axis” has been widely studied, several liver diseases including NAFLD are considered to be affected by the gastrointestinal environment determined by existing microorganisms [5].

*Helicobacter pylori* (*H. pylori*) infection is very common worldwide, especially in developing countries [6]. To the best of our knowledge, *H. pylori* infection has the greatest impact on the gastrointestinal environment, with approximately 50% of the global population estimated to be infected with *H. pylori* [7]. It has also been shown that the chronic infection with *H. pylori* may cause chronic atrophic gastritis [8]. There are many reports on the effects of *H. pylori* infection on various digestive organs [9–11]. Although *H. pylori*infection is the main cause of gastrointestinal diseases (i.e., chronic gastritis and peptic ulcer disease), a large number of studies have reported the association between extragastrointestinal diseases and *H. pylori* infection, such as neurological, metabolic, and allergic diseases [12]. For two decades, several studies have investigated the potential relationship between *H. pylori* infection and liver diseases of diverse etiologies [13].

NAFLD is thought to be associated with the development of *H. pylori* infection, but the results remain inconsistent [14–16]. A retrospective study conducted in Seoul involving 3663 health screening adults reported that NAFLD was not associated with *H. pylori* infection [15]. A similar conclusion was also observed in the Japanese population [14]. In contrast, a large-scale longitudinal study suggested that the association existed between *H. pylori* infection and NAFLD, and *H. pylori* could be a significant contributor to NAFLD pathogenesis [16]. It has also been demonstrated that *H. pylori* infection was an independent predictor of NAFLD in the general population of the United States. In addition, a study revealed that *H. pylori* eradication might prevent NAFLD by improving insulin resistance, atherosclerotic lipid abnormalities, and low inflammation [17]. Since the high prevalence of *H. pylori* infection and the difficult treatment of NAFLD, confirming the pathogenicity of *H. pylori* infection in NAFLD will undoubtedly provide insights into the new treatment strategy for NAFLD. Therefore, the current association between *H. pylori* infection and NAFLD remains worthy of further investigation.

We conducted a retrospective study to elucidate the seroprevalence of *H. pylori* infection and the association between NAFLD and *H. pylori* infection in north urban Chinese. This paper also explored the relationship between the severity of NAFLD (mild, moderate, and severe) and *H. pylori* infection, as well as the impact of MS on above relevance. The findings could help to determine the role of *H. pylori* infection in the pathogenesis of NAFLD, providing insights for novel treatment strategies for NAFLD.

## 2. Materials and Methods

### 2.1. Study Design and Participants

The retrospective study was performed at Aerospace Center Hospital in Beijing, China. All the subjects in this study were a healthy population who registered in the hospital health examination system between 2012 and 2015. Subjects without results of *H. pylori* infection status or abdominal ultrasound examinations were excluded. Information of demographic characteristics of all recruited subjects was collected, including height, weight, underlying diseases (hypertension, coronary heart disease, diabetes, and hyperlipidemia), and laboratory test results (low-density lipoprotein (LDL), high-density lipoprotein (HDL), triglyceride (TG), total cholesterol (TC), and abdominal ultrasound examination).

This study was carried out in accordance with the recommendations of the Ethical Committees of Aerospace Center Hospital. The protocol was approved by the Ethical Committees of Aerospace Center Hospital. All subjects gave written informed consent in accordance with the Declaration of Helsinki. The methods were carried out in accordance with the approved guidelines.

### 2.2. Diagnosis of *H. pylori* Infection and NAFLD

The *H. pylori* infection status was assessed by ELISA specific for anti-*H. pylori* IgG and IgM. The diagnosis of NAFLD was based on the results of abdominal ultrasonography. NAFLD and its classification were defined according to the Chinese Society of Hepatology. A confirmed diagnosis was based on the opinion of at least two well-trained doctors in the same hospital. Detailed classifications of NAFLD are as follows:

1. The near-field echo of the liver is diffusely enhanced (higher than the spleen and kidney), and the far-field echo is attenuated
2. The structure of the intrahepatic duct is not clear
3. The liver is enlarged, and the edge of the liver becomes dull
4. Color ultrasound showed that the color flow signal in the liver was reduced or not easy to display, but the blood vessels in the liver were normal
5. The right hepatic capsule and transverse echo showed unclear or incomplete

Subjects who present the first criterion plus one of the (2)-(4) criteria were diagnosed as “mild” fatty liver; while patients with (1) and any two of (2)-(4) were diagnosed “moderate” fatty liver; those with (1) and any two of (2)-(4) plus (5) were diagnosed as “severe” fatty liver.

### 2.3. Diagnosis of MS

Based on the diagnostic criteria of MS made by the Chinese Diabetes Society, meeting over three MS characteristics was diagnosed as MS. Details of MS characteristics are as follows: (1) body mass index (BMI) > 25 kg/m^2^ is defined as obesity; (2) TG concentration ≥ 150 mg/dL or use of TG-lowering medication is defined as high TG; (3) HDL − C concentration < 40 mg/dL in men and <50 mg/dL in women is defined as low HDL-C concentration; (4) systolic blood pressure ≥ 130 mmHg, diastolic blood pressure ≥ 85 mmHg, or use of antihypertensive medication is defined as hypertension; and (5) fasting glucose level ≥ 100 mg/dL, use of antidiabetic medication, or previously diagnosed type 2 diabetes is defined as blood glucose disorders.

### 2.4. Statistical Analysis

Descriptive statistics were performed, with continuous variables summarized as the mean and standard deviation and categorical variables summarized as the frequencies and proportions. The statistical significance between various groups was tested using the *χ*^2^ test for categorical variables and the independent *t*-test for continuous variables. A logistic regression model was used to calculate the association between NAFLD and *H. pylori* infection. Age, gender, underlying diseases, and MS were adjusted in the above model. We also analyzed the effects of NAFLD on *H. pylori* infection in different subgroups, i.e., age, gender, and number of MS characteristic. Odds ratios (ORs) and their 95% confidence intervals (CIs) were estimated. A two-sided *P* < 0.05 was considered statistically significant. All analyses were performed using Stata 12.0 (StataCorp LP, College Station, TX, USA).

## 3. Results

A total of 17971 subjects participated in the study, with the mean age of 45 ± 18 years old, and 5898 (32.8%) were female. Among these, 4.8% of subjects had diabetes, 23.4% had hypertension, 4.8% had coronary heart disease, 35.7% had hyperlipidemia, and 26.8% had NAFLD. BMI data were as follows: 41.8% subjects had normal weight, 30.9% were overweight, and 10.6% were obese. A total of 7803 (43.4%) subjects were positive for *H. pylori* infection. The prevalence of mild, moderate, and severe NAFLD was 20.7% (3726/7803), 4.1% (730/7803), and 2.1% (369/7803), respectively, in the subjects with *H. pylori* infection. Significant differences were detected in the variables of age, sex, hypertension, hyperlipidemia, NAFLD, LDL, HDL, TG, TC, BMI, and the number of MS characteristic (all with *P* < 0.05) between the subjects with and without *H. pylori* infection. All the basic characteristics are shown in Table 1.

The prevalence of NAFLD was associated with *H. pylori* infection (OR = 1.664; 95% CI: 1.549-1.787; *P* < 0.001). As the severity of NAFLD increased, the risk of *H. pylori* infection increased. Compared with those subjects without NAFLD, the risk of *H. pylori* infection in subjects with mild, moderate, and severe NAFLD increased by 60.7% (OR = 1.607; 95% CI: 1.487-1.736; *P* < 0.001), 77.0% (OR = 1.770; 95% CI: 1.519-2.063; *P* < 0.001), and 112.0% (OR = 2.120; 95% CI: 1.714-2.526; *P* < 0.001), respectively. All of the above results are shown in Table 2.

Subgroup analyses of factors affecting NAFLD development were performed to evaluate the consistency of the effect of *H. pylori* on NAFLD (Table 3). The results of subgroup analysis did not show the heterogeneity of risk of incident NAFLD from *H. pylori* infection besides subgroup of subjects without MS characteristic. Moreover, the risk of incident NAFLD from *H. pylori* infection increased in the subgroup of subjects meeting three or more than three MS characteristics, by comparison with the subgroup of those who meet one or two MS characteristics.

## 4. Discussion

We retrospectively evaluated the association between NAFLD and *H. pylori* infection in the Chinese population. Our study revealed that subjects at all NAFLD levels had a higher seroprevalence of *H. pylori* infection, and the severity of NAFLD was positively correlated with *H. pylori* infection. This association was still evident in the subgroup analysis besides subjects without MS characteristic.

Multiple studies have investigated the relationship between NAFLD and *H. pylori* infection, especially in Asia [18]. However, the conclusions were inconsistent and controversial. A large cohort study performed in Korea with 17028 participants showed that the participants with *H. pylori* infection were at a higher risk of NAFLD development compared to uninfected individuals [16], while a large-scale cross-sectional study of 13737 Japanese adults reported that *H. pylori* infection was not associated with NAFLD disease [14]. Moreover, there were two large cross-sectional studies carried out in South Chinese (Shanghai and Zhejiang) who underwent physical examination. The study conducted in Shanghai with 21456 subjects revealed that *H. pylori* infection was not independently associated with the risk of NAFLD in the total population [19], while the study conducted in Zhejiang with 20389 subjects suggested that the combination of *H. pylori* infection and WBC count was positively associated with NAFLD [20]. The discrepancy among previous findings might result from the geographical location, study design, and confounder adjustment. Recently, a systematic review and meta-analysis of 15 observational studies (published between 2013 and 2018) involving 97228 participants showed a robust result that existed a positive association between *H. pylori* infection and the risk of NAFLD [18].

Although so many researches have investigated the association between *H. pylori* infection and the risk of NAFLD, to our knowledge, this is the first large-scale retrospective study conducted in north urban Chinese, and it is suggested for the first time that the severity of NAFLD was positively correlated with *H. pylori* infection, which further validated that *H. pylori* infection was associated with the increased risk of NAFLD. The above epidemiological findings have also been supported by the studies of biological mechanisms. It has been reported that *H. pylori* infection can alter lipid profile [21], and NAFLD is closely related to abnormal lipid metabolism. Moreover, it is well accepted that inflammation is a central component of NAFLD pathogenesis [22]. A variety of inflammatory cytokines are involved in *H. pylori* infection, with the closest relationships detected among CRP, TNF-*α*, IL-6, and interleukin- (IL-) 1*β* [23, 24]. In addition, the formation of hepcidin in subjects with NAFLD increases the iron load and leads to iron-induced intestinal mucosal oxidative damage [25, 26], which in turn increases acid load [27]. It has been suggested that increased acid load can precipitate bile acids and reduce bile inhibition by *H. pylori*, thereby increasing the risk of *H. pylori* infection [27]. However, more direct evidence from epidemiological studies is needed to confirm this association, and the underlying mechanism should be confirmed with more studies.

MS could play essential roles and effects on the potential association between *H. pylori* infection and NAFLD. Multiple studies have investigated the relationship between *H. pylori* infection and MS, as well as NAFLD and MS. For example, a meta-analysis showed a significant positive correlation between *H. pylori* infection and MS, with a 34% higher risk of MS in *H. pylori*-infected individuals compared with noninfected individuals [28]. It has been reported that NAFLD is associated with abdominal obesity, hyperglycemia, hypertension, and dyslipidemia as each component of MS [29]. Moreover, several previous studies about the association between *H. pylori* infection and NAFLD adjusted MS characteristics as confounding factors to investigate whether NAFLD was a dependent factor of *H. pylori* infection [14, 19, 30]. In the subgroup analysis of this study, the association between *H. pylori* infection and MS was not significant in subjects without MS characteristic, while the association was stronger in the subjects meeting three or more than three MS characteristics than those who meet one or two characteristics. This is the first time that the subgroup analysis was performed based on the number of MS characteristic. It should be noted that the MS definition may alter across countries. For example, the large-scale cross-sectional study conducted in Japan also investigated the role of MS in the association of *H. pylori* infection and NAFLD, and the MS definition was based on Japanese criteria which defined visceral obesity as a necessary factor differently from Chinese criteria [14]. Thus, our current results should be considered with caution, and the effects of MS in different populations are worth further exploration.

Our research has some limitations. First, due to the limitation of retrospective studies, it was impossible to clarify causality. Second, serological testing for the presence of anti-*H. pylori* IgG and IgM did not indicate a current infection but only showed exposure to these bacteria, which may have biased the detection of *H. pylori* infection. Third, ultrasonography is not sensitive when steatosis occurs in less than 30% of hepatocytes. Finally, the study did not collect dietary factors from the study subjects, which requires further research.

## 5. Conclusion

We conducted a retrospective study based on the examination of a health record database with a large sample size to investigate the association between NAFLD and *H. pylori* infection. In conclusion, NAFLD may be associated with *H. pylori* infection. Younger, male NAFLD patients and those with more MS characteristics were more likely to have *H. pylori* infection.

## Figures and Tables

**Table 1:** Characteristics of the subjects in the study.

Variables	Total *N*^a^ = 17971	*H. pylori* (+) *n* = 7803	*H. pylori* (-) *n* = 10168	*P* value
Age (year), mean ± SD	45 ± 18	46 ± 18	44 ± 18	<0.001
<45	10525 (58.6)	4334 (55.5)	6191 (60.9)	<0.001
≥45	7446 (41.4)	3469 (44.5)	3977 (39.1)	
Sex, female (%)	5898 (32.8)	2374 (30.4)	3524 (34.7)	<0.001
Underlying diseases	5079 (28.3)	2260 (29.0)	2819 (27.7)	0.068
Hypertension	4202 (23.4)	1884 (24.1)	2318 (22.8)	0.034
Coronary heart disease	864 (4.8)	386 (4.9)	478 (4.7)	0.445
Diabetes	1950 (4.8)	852 (10.9)	1098 (10.8)	0.797
Hyperlipidemia	6412 (35.7)	2628 (33.7)	3784 (37.2)	<0.001
NAFLD	4825 (26.8)	2516 (32.2)	2309 (22.7)	<0.001
Mild	3726 (20.7)	1901 (24.4)	1825 (17.9)	<0.001
Moderate	730 (4.1)	407 (5.2)	323 (3.2)	
Severe	369 (2.1)	208 (2.7)	161 (1.6)	
LDL > 3.1 mmol/L	4801 (26.8)	2182 (28.1)	2619 (25.9)	0.001
HDL < 0.83 mmol/L	5254 (28.4)	2348 (30.1)	2906 (28.6)	0.027
TG > 1.71 mmol/L	8650 (48.1)	4074 (52.2)	4576 (45.0)	<0.001
TC> 5.7 mmol/L	2255 (12.6)	891 (11.4)	1364 (13.4)	<0.001
BMI (kg/m^2^), mean ± SD	24.0 ± 3.5	24.2 ± 3.5	23.9 ± 3.5	<0.001
Underweight	568 (3.2)	238 (3.5)	330 (3.7)	<0.001
Normal	7503 (41.8)	3107 (46.2)	4396 (49.8)	
Overweight	5560 (30.9)	2486 (37.0)	3074 (34.8)	
Obesity	1910 (10.6)	888 (13.2)	1022 (11.6)	
Number of MS characteristic				
0	2465 (13.7)	991 (12.7)	1474 (14.5)	<0.001
1-2	10402 (57.9)	4428 (56.7)	5974 (58.8)	
≥3	5104 (28.4)	2384 (30.6)	2720 (26.8)	

^∗^SD: standard deviation. ^a^Number of subjects.

**Table 2:** Odds ratios of *H. pylori* infection in different grades of NAFLD.

Variable	*n* (%)	*H. pylori*			
		Crude OR (95% CI)	*P*	^∗^Adjusted OR (95% CI)	*P*
NAFLD		1.620 (1.516-1.731)	<0.001	1.664 (1.549-1.787)	<0.001
No	13146 (73.2)	1.000		1.000	
Mild	3726 (20.7)	1.548 (1.439-1.666)	<0.001	1.607 (1.487-1.736)	<0.001
Moderate	730 (4.1)	1.873 (1.612-2.177)	<0.001	1.770 (1.519-2.063)	<0.001
Severe	369 (2.0)	1.920 (1.559-2.366)	<0.001	2.120 (1.714-2.526)	<0.001

^∗^Adjusted for age, gender, underlying diseases, and MS.

**Table 3:** The association between NAFLD and *H. pylori* infection in different subgroups.

Variable	Number of patients with fatty liver (%)	*H. pylori*			
		Crude OR (95% CI)	*P*	^∗^Adjusted OR (95% CI)	*P*
Age					
<45	2272 (21.6)	1.624 (1.479-1.783)	<0.001	1.679 (1.516-1.860)	<0.001
≥45	2553 (34.3)	1.534 (1.393-1.689)	<0.001	1.604 (1.449-1.776)	<0.001
Gender					
Female	1350 (22.9)	1.435 (1.269-1.621)	<0.001	1.418 (1.241-1.620)	<0.001
Male	3475 (28.8)	1.680 (1.551-1.818)	<0.001	1.761 (1.617-1.918)	<0.001
Number of metabolic syndromes					
0	175 (7.1)	1.068 (0.931-1.225)	0.347	0.964 (0.702-1.323)	0.819
1-2	2291 (22.0)	1.267 (1.121-1.431)	<0.001	1.233 (1.121-1.357)	<0.001
≥3	2359 (46.2)	2.535 (2.264-2.839)	<0.001	2.811 (2.497-3.163)	<0.001

^∗^Adjusted for age, gender, underlying diseases, and MS.

## Data Availability

The data used to support the findings of this study are available from the corresponding author upon request.
